# COVID-19 and the proliferation of urban networks for health security

**DOI:** 10.1093/heapol/czaa194

**Published:** 2021-01-25

**Authors:** Matthew R Boyce, Rebecca Katz

**Affiliations:** Center for Global Health Science and Security, Georgetown University, 3900 Reservoir Road NW, Room NW 306, Washington, DC 20057, USA; Center for Global Health Science and Security, Georgetown University, 3900 Reservoir Road NW, Room NW 306, Washington, DC 20057, USA

**Keywords:** Global health, governance, health security, policy, sustainable development, urban health

## Abstract

Recent years have witnessed cities establishing themselves as major players in addressing global issues, often taking collective action through international city networks and organizations. These networks are important, as they amplify the voices of municipal officials, who are often excluded from high-level decision-making, and can also provide a platform for officials from low- or middle-income nations to participate in higher-level political forums. The global response to the COVID-19 pandemic has included traditional public health stakeholders—including supranational organizations, international non-governmental organizations and national authorities—but has also featured mayors and city networks, in an unprecedented fashion. Existing networks without an explicit focus on health have shifted their focuses to prioritize pandemic response and several new networks have been created. These developments are significant, not only because they represent a shift in health governance and policy, but also because cities and urban networks more broadly have exhibited a nimbleness and pragmatism unmatched by higher levels of governance. These characteristics could prove beneficial for addressing the current pandemic, as well as future health issues and emergencies. Furthermore, given the relative lack of engagement with health security issues before the COVID-19 pandemic, the drastic health and economic impacts associated with it, and the demonstrable value added by strong city leadership, there are an open policy window and a compelling case for continued city engagement in health security.

**KEY MESSAGES**

- Cities have demonstrated an aptitude and nimbleness in responding to the COVID-19 pandemic that has proved useful and addressed the shortcomings of higher levels of governance.
- Municipal authorities have recently established themselves as major actors in global issues—often acting collectively through inter-city networks and organizations—but historically, these networks have not engaged substantively on health-related issues.
- The pandemic has shifted or altered the focus of several existing networks and galvanized the creation of several new networks focused on preparing and responding to the public health emergency.
- There is an open-window for sustained local-level engagement with health security issues that could have implications for better preparing for and responding to future public health emergencies.

The rapid spread of the SARS-CoV-2 virus has prompted a collective, global response to the resulting COVID-19 pandemic. This response has included traditional public health stakeholders—supranational organizations (e.g. the World Health Organization), international non-governmental organizations (e.g. Médecins Sans Frontières) and national authorities (e.g. ministries of health, national public health institutes)—but has also featured collective action by sub-national authorities, such as mayors and municipal authorities, in an unprecedented fashion.

In recent years, cities have established themselves as major players in addressing transnational issues, such as global warming and climate change, health and pervasive socioeconomic inequalities (Acuto, 2013). While a handful of global cities maintain the clout to influence national and international agendas on their own, to a growing extent, they have sought to do so primarily by forming and operating through organized networks. While this phenomenon is not new—as the first formal international city organization was established over a century ago—our world has never seen such a proliferation of city networks to address global issues (Fernández de Losada and Abdullah, 2019). Indeed, by some estimates, over 200 formal city networks—defined as formalized organizations, primarily composed of cities, and characterized by established patterns of communication, policy‐making and exchange—exist globally, and thousands of para-diplomatic connections further define the relationships between cities (Acuto and Rayner, 2016). While some of these networks have domestic focuses, many more are truly global—amplifying the voices of municipal authorities in low- or middle-income nations that otherwise may be lost to the forces of western dominated politics and norms. However, the utility of these networks is not exclusively confined to advocacy and influencing political agendas, but also extends to more practical considerations, such as taking strategic joint-action and sharing lessons, models and best practices. These developments are significant, not only because they represent a shift in the health governance and policy, but also because cities and urban networks more broadly have exhibited a nimbleness unmatched by more bureaucratic forms of governance that could prove beneficial for addressing future health issues and emergencies.

We assume that there are advantages to city networking—namely those previously mentioned—that render them a valuable contribution to pandemic response. In this commentary, we provide a brief but substantiated overview of how city networks have contributed to the COVID-19 pandemic, pose questions and assert that there is a compelling need for the continued engagement of city networks in health security.

## Pandemic pivots of existing urban networks

A 2018 review of 99 city networks found that a majority of them had a specific or very narrow focus (Foster and Swiney, 2018). Of this majority, most networks sought to address environmental issues (e.g. climate change, green infrastructure and transportation, environmental sustainability) and health was identified as a neglected theme. Those networks that did prioritize health, such as the World Health Organization’s European Healthy Cities Network, focused primarily on developing urban spaces to promote good health, addressing socioeconomic inequalities and determinants of health, or vague goals related to achieving health and well-being [World Health Organization Regional Office for Europe (WHO EURO), 2020].

It was within this context that, in 2018, the Global Parliament of Mayors (GPM) took up the cause of pandemic preparedness and prioritized it as a key component of urban health planning and committed to developing an intra-city mechanism to share information and experiences during an emergency response (Global Parliament of Mayors, 2018). In doing so, they became one of the first, if not the first, mayoral networks to explicitly prioritize preparing for responding to infectious disease threats.

The GPM is now in good company. Many other networks, including many that did not prioritize health, have pivoted their focuses to address the COVID-19 pandemic. For example, the C40 Network, a group of 96 cities dedicated to addressing climate change, has assembled a regularly updated knowledge hub with resources, articles, policy briefs and indices to help cities better understand and respond to the pandemic. They have also hosted virtual meetings of mayors to share experiences in responding to the pandemic and created a COVID-19 Recovery Task Force to promote a fair and sustainable recovery (C40 Cities, 2020). However, mayors have also used the connections in the network to bolster public health capacities. For example, the City of Seattle leveraged its relationship with the City of Seoul, which was established through the C40 Network, to secure additional diagnostic tests as a means of improving access to COVID testing within the city (Anderson and De Jong, 2020).

Similar actions have been taken by both international networks—such as the EuroCities Network (EuroCities, 2020), the Global Resilient Cities Network (formerly the 100 Resilient Cities Program) (Global Resilient Cities Network, 2020), the Metropolis Network (Metropolis, 2020) and the World Organization of United Cities and Local Governments [The World Organization of United Cities and Local Governments (UCLG), 2020]—and domestic networks—such as the National League of Cities (National League of Cities, 2020) and the United States Conference of Mayors [United States Conference of Mayors (USCM), 2020].

## New mayoral networks for pandemic response

In addition to the shifting focus of existing mayoral networks, the COVID-19 pandemic has spurred the creation of new networks, focused exclusively on responding to the public health emergency or improving the response to future events.

Bloomberg Philanthropies has partnered with the National League of Cities, the United States Conference of Mayors and several academic institutions to create a new network—the COVID-19 Local Response Initiative—to help cities in the USA address the pandemic. Members of this network participate in weekly conference calls and daily e-mail updates are sent to all cities involved with the latest actions that member cities are taking to fight the pandemic, mobilize the public and support the local economy (Bloomberg, 2020).

Similarly, the Rockefeller Foundation has created an ad hoc city network called the Testing Solutions Group (TSG), i.e. focused on facilitating the exchange of lessons and best practices between city public health officials and linking officials with public health experts to scale up COVID-19 testing while safeguarding the health of communities (Rockefeller Foundation, 2020). In addition to the peer-to-peer network, the TSG convenes experts to develop knowledge products (i.e. strategy and policy recommendations) for local authorities, help city officials collect real-time data to promote evidence-based testing strategies and provide technical and financial assistance for enhancing testing in vulnerable populations.

The WHO has also discussed creating a Global Cities Network for Health Security that will be a part of their larger Global Strategic Preparedness Network. This network would be comprised of mayors and other local authorities and would strive to document, share and learn from the experiences among cities in advancing health security. The WHO has also considered allowing these authorities to advocate at future political-level meetings—another request of the GPM in 2018 (Global Parliament of Mayors, 2018). While time will only tell if these larger efforts will come to fruition, they have released specific interim COVID-19 preparedness guidance for local authorities in cities and urban environments that, at a minimum, acknowledge the important role cities have played in the response to this global crisis [World Health Organization (WHO), 2020].

## The future of mayoral networks in health security

The successful response to public health emergencies requires strong, evidence-based leadership. Some have argued that cities currently are the most powerful they have been since city-states dominated during the Renaissance (Swiney and Foster, 2019); and indeed, mayors and other city officials have stepped up to fill the gaps left by some higher levels of governance to provide consistent and decisive public-sector leadership from the bottom-up (Anderson and De Jong, 2020).

But the future of mayoral networks in health security is opaque and likely to be influenced by a variety of factors. For instance, the involvement of cities and city networks in health security will be influenced by larger tensions in health policy and governance—especially concerning powers and authorities relating to outbreak response. This has been seen in a variety of cities throughout the COVID-19 pandemic, such as Atlanta (Bogel-Burroughs and Robertson, 2020), Madrid (Dombey, 2020), Manchester and Marseilles (The Economist, 2020).

The involvement of city networks in health security also begs two major questions: first, is this recent trend beneficial; and second, are efforts sustainable in the long-term? The city network ecosystem has already been characterized as saturated, competitive (as opposed to complementary) and at risk of being duplicative—all of which could limit efficiency and waste limited resources (Fernández de Losada and Abdullah, 2019). Regarding long-term sustainability, pandemic preparation is notorious for suffering from cycles of panic and neglect in which efforts are increased when a threat is imminent—when it is too late for them to have optimal impacts—and then quickly decreased when the threat subsides [Global Preparedness and Monitoring Board (GPMB), 2019]. Furthermore, this second question relates not only to financing for pandemic preparedness but also with regard to superficial or temporary involvement of cities in international health security agendas, which can lead to frustration and eventual disengagement.

It seems unlikely that networks with other stated priorities and agendas will continue to prioritize health security once the pandemic subsides. However, given the relative dearth of local-level attention or engagement with health security issues prior to the pandemic, the drastic health and economic impacts associated with it, and the value demonstrated by strong city leadership and engagement, we feel there is a clear and compelling case for continued city engagement in health security. Ultimately, it seems unlikely that it will be possible to talk about health security or sustainable development issues without including cities and as a part of the discussion following the COVID-19 pandemic.

## Funding

This work did not receive funding support.

*Conflict of interest statement*. None declared.

*Ethical approval*. This work did not include research with human subjects and thus did not require ethical clearance as outlined in the World Medical Association's Declaration of Helsinki.
